# Drug use pattern using WHO core drug use indicators in public health centers of Dessie, North-East Ethiopia

**DOI:** 10.1186/s12911-021-01530-w

**Published:** 2021-06-25

**Authors:** Teklehaimanot Fentie Wendie, Abdulmejid Ahmed, Solomon Ahmed Mohammed

**Affiliations:** 1 Department of Pharmacy, College of Health Science, Wollo University, Dessie, Ethiopia; 2 Pharmacy Student, Wollo University, Dessie, Ethiopia

**Keywords:** Prescribing indicator, Patient care indicator, Health facility indicator, Ethiopia

## Abstract

**Background:**

Rational drug use requires that patients receive and take medication appropriately. Though the process of diagnosis and pharmaceutical care is complex, World Health Organization (WHO)/international network for rational use of drugs (INRUD) core drug use indicators investigate drug use to minimize the hazardous effect of the drug and enhance the wise use of scares resources. This study assessed drug use patterns in health centers of Dessie town using WHO/INRUD indicators.

**Methods:**

A cross-sectional study was conducted in public health centers of Dessie town. Data were retrospectively collected from 1500 prescriptions dispensed from January 1, 2018, to December 31, 2018 using WHO data collection tool to assess prescribing indicators. For patient care and health facility indicators, 600 patients and 3 health centers were prospectively reviewed. Systematic random sampling was used to select samples. Data were analyzed using the Statistical Package for the Social Sciences version 20.

**Results:**

The average number of drugs per encounter was 2.1. The percentage of encounters with antibiotics and injection was 44% and 13.9%, respectively. The percentage of drugs prescribed by generic name and from an essential drug list was 98% and 100%, respectively. On average, patients spent 4.7 min for consultation and 105 s for dispensing. From 1305 prescribed drugs, 92% were dispensed, while only 4% were labeled adequately. More than half (54.8%) of patients had adequate knowledge of their medication. None of the health centers had an essential drug list. The availability of key essential medicines was 64.10%.

**Conclusion:**

This study demonstrated irrational drug use practices in all healthcare facilities. Polypharmacy, antibiotics over-prescribing, short consultation and dispensing times, inadequate labeling of medicines, inadequate level of patients' knowledge about prescribed medicines, and unavailability of key drugs in stock were found to be the major problems. Continuous refreshment trainings on rational use of drugs and WHO recommendations should be given for prescribers and pharmacists. Further, we recommend studies involving large number of facilities to estimate overall prescribing practices.

## Background

Rational drug use requires that patients get medications that meet their clinical needs, in appropriate doses, for an adequate duration and at an affordable cost [1]. It minimizes the incidence of adverse drug events and maximizes the benefits that can be derived from the optimal use of medications. Rational use of drugs can also result in optimal use of scares health care resources [1–3].

Common types of irrational medicine use are: polypharmacy, frequent use of antibiotics, inadequate dosage, over-use of injections when oral formulations would be more appropriate, failure to prescribe in accordance with clinical guidelines, prescribing by brand names and inappropriate self-medication [4, 5]. Inappropriate drug prescribing, dispensing and use is a global burden [5] though the magnitude of the problem was reported to be higher in developing countries [6]. Various studies were conducted to investigate rational drug use across the world. Deviations from the WHO recommendations were reported in Nepal [7], Uzbekistan [8], Saudi Arabia [9], Nigeria [2], Zambia [10], Kenya [11] and Ethiopia [6, 12–17].

Irrational use of drugs result in treatment failure, adverse drug events and increased cost on patients and society [18]. Exposure to multiple drugs is associated with increased risk of adverse drug reactions [19, 20]. Indiscriminate utilization of antibiotics also results in drug resistance and the need for other alternatives which may not be available or affordable, and later results in loss of patient confidence in the health care system [21]. Globally, 16 billion injections are administered every year [22]. In developing countries, infections resulting from the use of unsterile syringes and needles are a terrible self-inflicted disaster [23]. In many cases these injections are unnecessary and can be replaced with oral medicines [22, 23]. Unsafe injections have the potential to transmit infections including hepatitis B virus, hepatitis C virus and human immunodeficiency virus. According to a mathematical modelling study published in 2014, the number of infections associated with unsafe injections were 1.67 million, 315,120 and 33,877 for Hepatis B Virus, Hepatitis C Virus and Human Immunodeficiency Virus, respectively [24].

Due to the complex nature of pharmaceutical care process, there should be an essential tool which investigates drug use pattern in health facilities [25]. In 1985, WHO organized a conference in Nairobi and developed core and complementary drug use indicators [1]. Core drug use indicators are more informative and feasible, less likely to fluctuate over time and place, and provide a simple tool for quickly and reliably assessing drug use than complementary indicators. Thus, they have been selected for better quantitative evaluation of rational drug use [5, 26].

So far, three major types of core drug use indicators are available; prescribing indicators (average number of drugs in prescriptions, percentage of drugs prescribed by generic name and facility specific medicine list, percentage of encounters with an antibiotic and injection prescribed); patient care indicators (average consultation and dispensing time, percentage of drugs dispensed, adequately labeled, and knowledge of patients’ on dosage), and health facility indicators (availability of essential drugs list and key drugs) [26].

Prescribing indicators utilize prescriptions to measure the performance of health care providers related to the appropriate use of drugs. They can be measured with in a defined geographic area either to describe drug use at a given point in time or to monitor changes over time using retrospective or prospective methods [25, 26]. Prescriptions with many drugs are a signal for inappropriate prescribing and an important index of the scope for intervention in rationalizing of prescribing practices [27]. Patient care indicators assess what patients experience at the health facility and how well they know about the pharmaceuticals prescribed and dispensed to them [25, 26]. Hence, based on an evaluation, ways for achieving rational and cost-effective medication use may be suggested [18]. Therefore, the purpose of this study was to investigate drug use pattern in a reproducible manner by employing the WHO/INRUD core drug use indicators at selected public health centers in Dessie town.

## Methods

### Study area and period

The study was conducted from February 10 to April 10, 2019 at three public health centers in Dessie town, North-East Ethiopia. Dessie is located 401-kilo meters away from Addis Abba, the capital of Ethiopia. In Dessie, eight public health centers are serving for 151,094 populations.

### Study design

A cross-sectional study was conducted.

### Population

WHO/INRUD prescribing indicators: All prescriptions dispensed from the out-patient pharmacy of each health center were taken as the source population; however, only those prescriptions dispensed from January 1, 2018 to December 31, 2018 were taken as study populations.

WHO/INRUD patient care indicators: All patients who visited and treated in the outpatient departments of each health center from February 10 to April 10, 2019, were the source population. Patients with prescriptions containing at least one drug and fulfil the inclusion criteria were eligible for enrolment in the study.

WHO/INRUD health facility indicators: All pharmacy professionals and all essential drugs were the source population. Pharmacy department heads who were in charge to deliver pertinent information and the WHO model list of key drugs were the study population.

### Eligibility criteria

Those prescriptions which were legible and complete were included for prescribing indicators. Patients aged above 18 years and volunteer to participate were included for patient care indicators. Illegible and incomplete prescriptions as well as those prescriptions that contain only medical supplies were excluded. Patients visiting each health center outside the normal working hours, who were unconscious and/or had hearing problem were also excluded from the study. Five health centers were excluded because of unavailability of well-organized records due to different reasons like mix-ups and/or lose while changing stores. Thus, Dessie health center (DHC), Segno-gebeya health center (SGHC) and Buanbua-wuha health center (BWHC) were included in the study.

### Sample size determination and sampling techniques

Three public health centers were conveniently selected. The sample size for assessing prescribing indicators was based on WHO recommendation where at least 600 encounters should be included in the survey. By taking the retrospective nature of the study and the number of health facilities included in to consideration, a total of 1500 prescriptions (500 prescriptions per health center) were selected by systematic random sampling from 37,800 prescriptions dispensed over a year and tied on a daily basis by taking every 50 prescriptions in DHC, every 25 prescriptions in SGHC, and BWHC.

At least 100 cases per health facility are recommended by WHO for WHO/INRUD patient care indicators [26]. In this study, a total of 600 patients (200 patients/health center) were included. Simple random sampling technique was used to recruit study participants. Moreover, pharmacy heads in selected health facilities were included for WHO/INRUD health facility indicators assessment as they were supposed to be rich in key information.

### Study variables

The dependent variables of the study were WHO prescribing, patient care and health facility indicators [26]. Socio-demographic characteristics (age and gender) of the study participants were predictor variables.

*The average number of drugs prescribed per encounter*: the total number of different medicines prescribed and divided by the number of encounters. Combinations drugs prescribed for treating one health problem were counted as one.

*Percentage of drugs prescribed by generic name*: the summation of number of drugs prescribed by generic name was divided by the total number of drugs prescribed, and multiplied by 100.

*Percentage of encounters with an antibiotic prescribed*: the summation of number of patient encounters treated with antibiotic divided by the total number of encounters, and multiplied by 100.

*Percentage of encounters with an injection prescribed*: the summation of number of patient encounters treated with an injection divided by the total number of encounters, and multiplied by 100.

*Percentage of medicines prescribed from essential drugs list:* the total number of medicines prescribed from list of essential medicines divided by the total number of medicines prescribed, and multiplied by 100.

*Average consultation time*: the total time for a series of consultations, was summed and divided by the number of consultations.

*Average dispensing counseling time*: the total time for counseling series of encounters was summed and divided by the number of encounters observed.

*Percentage of drugs actually dispensed:* the summation of the number of drugs dispensed at the health facility was divided by the total number of drugs prescribed, and multiplied by 100.

*Percentage of drugs adequately labeled:* the number of medicines adequately labeled was summed and divided by the total number of medicines dispensed, multiplied by 100.

*Patients’ knowledge of correct dosage*: the total number of patients who can adequately report the dose, frequency, and duration/total quantity dispensed for all medicines divided by the total number of patients and multiplied by 100.

*Availability of copy of essential drugs list*: the degree to which copies of the facilities medicine list is available (yes or no) at health facilities.

*Availability of key drugs*: the total number of key medicines available in stock divided by the total number of medicines on the checklist, and multiplied by 100.

*Availability of standard treatment guidelines (STG)*: the degree to which copies of STG is available (yes or no) in the health facility.

### Operational definition

*Combination of drugs*: Two or more drugs which are prescribed for treating a given health problem.

*Dispensing time*: is the length of the time between the patient gives the prescription to the dispenser and leaves the dispensary.

*Labeling*: the label is adequate if; the generic name of drug, strength, dose, quantity dispensed, frequency of administration, direction for use, expiry date, name of the patient, name, storage conditions, and special precautions are written.

*Prescription*: a paper which contains drug order and issued to a patient to collect drugs from the dispensing unit.

### Data collection tool and procedure

A structured data collection tool adopted from the WHO core medicine use indicator was used to collect data [26]. The interview questionnaire comprised questions related to socio-demographic features followed by questions that assessed the knowledge of patients about the dosage regimen of their medication including when and in what quantity to be taken, the storage condition and cautions' to be taken. Pretest on the data collection tools was conducted in 5% of samples of Dessie Referral Hospital. The data were collected by three trained pharmacists under the supervision of the principal investigators. Prescribing indicator data were retrospectively extracted from prescriptions of the past one year. Information on patient care and health facility indicators were collected prospectively. Data were checked for completeness, accuracy and consistency immediately after collection and appropriately arranged and kept in a secured place for compilation and analysis. The WHO guideline was carefully applied to ensure data reliability.

### Data processing and analysis

Data entry and analysis was done using the Statistical Package for the Social Sciences version 20. Descriptive statistics results were presented in the form of mean and percentage. Comparisons between individual facilities and prescribers were not made since sample size drawn from each health facility or per prescriber must be > 30 [26].

Drug use pattern was comprehensively assessed using WHO core drug use indicators [26]. Moreover, an index for each indicator was used for a comprehensive appraisal of medical care [5]. Number of drugs per prescriptions, antibiotic, and injection use indices were calculated by dividing the optimal level to the observed value. Others indices (index of prescribing by generic name, index of prescribing from Essential Drugs List (EDL), index of consultation, and dispensing time, index of drugs dispensed, and labeled, index of patients' knowledge on key drug aspects, index of EDL, and essential drugs availability) was computed by dividing the observed value by optimal level. The optimal values for all indicators were adopted from WHO [25, 28].

One is defined as the optimal index for all indicators. The value closer to 1 indicates the better rational drug use. The summation of the index values of all prescribing indicators resulted Index of Rational Drug Prescribing (IRDP). Then, the Index of Rational Patient-Care Drug Use (IRPCDU) and the Index of Rational Facility. Specific Drug Use (IRFSDU) was calculated. The value of IRDP, IRPCDU, and IRFSDU was summed to have Index of Rational Drug Use (IRDU). Then, health facilities were ranked according to the overall indices score. The higher IRDU value is an indicator of good rational drug use [5].

## Results

### WHO/INRUD prescribing indicators

A total of 1500 patient prescriptions were assessed retrospectively in the medical outpatient pharmacy of health centers. A total of 3199 drugs were prescribed with an average number of 2.1 drugs per prescription. The total numbers of drugs prescribed by generic name were 3137 (98%). An antibiotic was prescribed in 660 (43.9%) encounters and an injection was prescribed in 209 (13.9%) encounters. All the drugs (100%) prescribed were on the EDL of Ethiopia (Table 1).

**Table 1:** WHO/INRUD prescribing indicators in health centers of Dessie town from January 1, 2018 to December 31, 2018 (N = 1500)

Prescribing indicators	SGHC	DHC	BWHC	Total	WHO standard
Average number of drugs per encounter	1069 (2.13)	1018 (2.03)	1112 (2.2)	3199 (2.1)	(1.6%–1.8)
Percentage of encounter with antibiotics	222 (44.4)	241 (48)	197 (39.4)	660 (44)	(20.0–26.8)
Percentage of encounters with injection	58 (11.6)	82 (16.4)	69 (13.8)	209 (13.9)	(13.4–24.1)
Percentage of drugs prescribed by generic name	1047 (98)	1008 (99)	1082 (97)	3137 (98)	100
Percentage of drugs from essential drug list	100	100	100	100	100

SGHC, Segno Gebeya Health Center; DHC, Dessie Health Center; BWHC, Buanbua Wuha Health Center

On average 2.10 drugs were prescribed in health centers of Dessie town and 652 (43.4%), 388 (25.8%), and 362 (24.1%) of the prescriptions had two, one, and three drugs respectively (Table 2).

**Table 2:** Number of drugs per encounter in health centers of Dessie town from January 1, 2018 to December 31, 2018 (N = 1500)

No of drugs	SGHC	DHC	BWHC	Total	WHO standard
One	128 (25.60)	122 (24.40)	138 (27.60)	388 (25.80)	(1.6%–1.8)
Two	206 (41.20)	231 (46.20)	215 (43.0)	652 (43.40)	
Three	141 (28.20)	103 (20.60)	118 (23.60)	362 (24.10)	
Four	20 (4)	17 (3.40)	37 (7.40)	74 (4.90)	
Five	4 (0.80)	9 (1.80)	6 (1.20)	19 (0.80)	
Six	1 (0.20)	2 (0.40)	2 (0.40)	5 (0.30)	
Total	1069 (2.13)	1018 (2.18)	1112 (2.22)	3199 (2.10)	

SGHC, Segno Gebeya Health Center; DHC, Dessie Health Center; BWHC, Buanbua Wuha Health Center

The most commonly prescribed antibiotics were amoxicillin 137 (20.75%), Ciprofloxacin 106 (16%), Doxycycline 99 (15%), Norfloxacin 75 (11.4%), Cloxacillin 74 (11%), Ampicillin 48 (7.3%), and Cotrimoxazole 44 (6.7%) (Table 3).

**Table 3:** Antibiotics prescribed in health centers of Dessie town from January 1, 2018 to December 31, 2018 (N = 660)

Antibiotics	SGHC	DHC	BWHC	Total (percentage)
Amoxicillin	47 (21.17)	51 (21.16)	39 (19.80)	137 (20.8)
Ciprofloxacin	36 (16.22)	38 (15.77)	32 (16.24)	106 (16)
Doxycycline	34 (15.32)	37 (15.35)	28 (14.21)	99 (15)
Norfloxaciline	27 (12.16)	23 (9.54)	25 (12.69)	75 (11.4)
Cloxacillin	20 (9.01)	31 (12.86)	23 (11.68)	74 (11.2)
Ampicillin	14 (6.31)	18 (7.47)	168.12	48 (7.3)
Cotrimoxazole	12 (5.41)	21 (8.71)	11 (5.58)	44 (6.6)
Others^a^	32 (14.41)	22 (9.13)	23 (11.68)	77 (11.7)

SGHC, Segno Gebeya Health Center; DHC, Dessie Health Center; BWHC, Buanbua Wuha Health Center

^a^Benzathine penicillin G, Gentamycin, Erythromycin, Clarithromycin, Tetracycline, Chloramphenicol, Ceftriaxone, and Cephalexin

### WHO/INRUD patient care indicators

Half of the respondents were females. With regards to age composition, 43 (40.5%) and 229 (38.17%) were within the age groups of 9–44 and 45–64 respectively (Table 4).

**Table 4:** Socio-demographic characteristics of respondents in health centers of Dessie town, 2019 (N = 600)

Socio-demographic characteristics	SGHC	DHC	BWHC	Total
Sex				
Male	75 (37.5)	118 (59)	102 (51)	295 (49.17)
Female	125 (62.5)	82 (41)	98 (49)	305 (50.83)
Age				
19–44	85 (42.5)	85 (42.5)	73 (36.5)	243 (40.5)
45–64	76 (38)	63 (31.5)	90 (45)	229 (38.17)
≥ 65	39 (19.5)	52 (26)	37 (18.5)	128 (21.33)

SGHC, Segno Gebeya Health Center; DHC, Dessie Health Center; BWHC, Buanbua Wuha Health Center

The average consultation and dispensing times were 4.7 min and 105 s. From 1305 total prescribed drugs, 1203 (92%) were actually dispensed, off this 49 (4%) were adequately labeled (Table 5). More than half (54.8%) of patients had adequate knowledge of their medication, while 271 (45.2%) patients had inadequate knowledge.

**Table 5:** WHO/INRUD patient care indicators in health centers of Dessie town, 2019 (N = 600)

Patient care indicators	SGHC	DHC	BWHC	Total	WHO standard
Average consultation time (minute)	5.5	4.2	4.3	4.7	10 min
Average dispensing time (second)	92	120	104	105	> 180 s
Number of drugs prescribed	435	442	428	1305	
Percentage of medicines actually dispensed	392 (90.1)	414 (91.5)	399 (93)	1203 (92)	100%
Percentage of medicines adequately labeled	0	0	0	0	100%

SGHC, Segno Gebeya Health Center; DHC, Dessie Health Center; BWHC, Buanbua Wuha Health Center

Among 1203 dispensed drug, frequency of administration was mentioned on the drug label in 373 (31%), the dose was written in the label in 364 (30.3%), quantity dispensed were written in 38 (3.1%), 128 (10.6%) of the direction of the drug for use were written, and 52 (4.3%) drugs strength were mentioned on the drug labeled. However, patient name, generic name, quantity dispensed, storage condition, and expiry date were not written on the drug label of dispensed drugs (Table 6).

**Table 6:** Labeling status of dispensed drugs in in health centers of Dessie town, 2019 (N = 1203)

Labeled component	SGHC	DHC	BWHC	Total	WHO standard
Patient name	–	–	–	–	100%
Generic name	–	–	–	–	
Strength	18 (1.4)	12 (0.9)	22 (1.8)	52 (4.3)	
Dose	124 (10.3)	103 (8.5)	137 (11.38)	364 (30.3)	
Quantity dispensed	10 (0.8)	13 (10.8)	15 (1.2)	38 (3.1)	
Frequency of administration	128 (10.64)	106 (8.8)	139 (11.5)	373 (31)	
duration of treatment	23 (1.9)	85 (7.0)	20 (1.6)	128 (10.6)	
Storage condition	–	–	–	–	
Expire date	–	–	–	–	

SGHC, Segno Gebeya Health Center; DHC, Dessie Health Center; BWHC, Buanbua Wuha Health Center

### WHO/INRUD health facility indicators

None of the health centers in the current study had its own EDL or STG. The percentage availability of the WHO model list of key essential medicines was ranged from 61.54 to 69.23 (Table 7). The overall availability of the WHO model list of key essential medicines was 64.10%.

**Table 7:** Availability of key essential drugs in health centers of Dessie town, 2019 (N = 13)

Key essential medicines	SGHC	DHC	BWHC
Oral rehydration salt	✓	✓	✓
Sulfamethoxazole + trimethoprim tablet	✓	✓	✓
Paracetamol tablet	✓	✓	✓
Procaine penicillin injection	–	✓	–
Chloroquine tablet	–	–	–
Ferrous salt + folic acid tablet	✓	✓	–
Mebendazole tablet	✓	✓	✓
Iodine	–	–	✓
Tetracycline eye ointment	✓	✓	✓
Gentian violate	✓	✓	–
Benzoic acid + salicylic acid ointment	–	–	✓
Acetyl salicylic acid tablet	✓	✓	✓
Vitamin A	–	–	–
Percentage of essential drugs availability	61.54	69.23	61.54
WHO standard	100%		

SGHC, Segno Gebeya Health Center; DHC, Dessie Health Center; BWHC, Buanbua Wuha Health Center

Heath centers of Dessie town had an IRDU value of 7.58. Among health centers, BWHC represented the highest rank for IRDP. The highest rank for IRPCDU was represented by SGHC and DHC while the highest rank for RFSDU was scored by DHC (Table 8).

**Table 8:** Index of WHO/INRUD drug use indicators in health centers of Dessie town from January 1, 2018 to December 31, 2018 (N = 1500)

Indicators	IRDU	SGHC	DHC	BWHC	Total
Prescribing indicators	Non-polypharmacy index	0.84	0.88	0.81	0.85
	Generic name index	0.98	0.99	0.97	0.98
	Rational antibiotic index	0.6	0.55	0.68	0.6
	Injection safety index	1	1	1	1
	EDL index	1	1	1	1
	IRDP	4.42	4.42	4.46	4.43
	Rank	2	2	1	
Patient-care indicators	Consultation time index	0.55	0.42	0.43	0.47
	Dispensing time index	0.51	0.6	0.57	0.58
	Dispensed drugs index	0.9	0.91	0.93	0.92
	Labeled drugs index	0	0	0	0
	Patients’ knowledge index	0.55	0.58	0.51	0.54
	IRPCDU	2.51	2.51	2.44	2.51
	Rank	1	1	2	
Facility-specific indicators	Index of EDL	0	0	0	0
	Index of key drugs in stock	0.61	0.69	0.61	0.64
	IRFSDU	0.61	0.69	0.61	0.64
	Rank	2	1	2	
Grand total	IRDU	7.54	7.62	7.51	7.58
	Rank	2	1	3	

SGHC, Segno Gebeya Health Center; DHC, Dessie Health Center; BWHC, Buanbua Wuha Health Center

## Discussion

Despite the selection of essential medicines, patients should receive the right medicine, in an adequate dose for an adequate duration, with appropriate information and follow-up treatment, and at an affordable cost [5, 25]. The behavior of health care providers related to the appropriate use of drugs could be investigated by WHO/INRUD core drug use indicators which could help to minimize the hazardous effect of the drug and enhance the wise use of scares resources. The present study assessed the specific aspects of drug use pattern in a reproducible manner by employing the WHO/INRUD core drug use indicators.

In this study, the average number of drugs per encounter was 2.1. The value was slightly higher than the WHO standard (1.6–1.8) [5, 26]. Moreover, the value was also higher than the findings from South Ethiopia (1.9) [12], North Ethiopia (1.96) [17], East Ethiopia (1.9) [28], and West Shoa zone (1.74) [29]. However, this was in line with the value reported from Nekemte helth center (2.1) [16] and less than that of a study conducted in North East Ethiopia (2.5) [30], North West Ethiopia (2.4) [13, 15], and public hospital of Eastern Ethiopia (2.34) [31]. Lower findings were also reported from Sierra Leone (4.37) [32], Kenya (2.9) [11], Pakistan (3.4) [33], Zambia (2.4) [10], and Egypt (2.5) [34]. Polypharmacy might be attributed to prescribers' incompetency, unavailability of clinical practice setup, financial incentives to the prescribers, and the shortage of essential medicines. The incidence of adverse drug reactions will increase upon exposure to multiple drugs and impose an economic burden on patients and society. Keeping the mean number of drugs per encounter low is recommended to minimize the risk of development of drug resistance, drug interaction, adverse effect of the drug as well as related expenditure [18].

The percentage of encounters with antibiotics was 44% which was higher than the WHO standard value of 20.0—26.8% [5, 26]. It was higher than the antibiotics utilization in East Ethiopia (37.5%) [28] and North East Ethiopia (34.64%) [30]. But, it was lower than reports from Nekemte health center (69.1%) [16], South Ethiopia (58.1%) [12], North Ethiopia (58.6%) [20], West Shoa zone (48.9%) [29] and North West Ethiopia (71.36%) [13]. The finding was also much lower than the findings from Sierra Leone (71.5%) [32], Kenya (84.8%) [11], Pakistan (48.9%) [33], Zambia (65.4%) [10] and Eritrea (53%) [35]. The high percentage of antibiotics prescribed might be due to the empiric nature of the treatment approach, low level of prescribers' knowledge about the risk of antimicrobial resistance and the side effect of antibiotics or patient preference. The overuse of antibiotics for empirical treatment is an indication of a problem that could facilitate the emergence of resistance [8]. Irrational usage of antibiotics increases the risk for the antimicrobial resistance leading to increased morbidity, mortality and economic burden on health-care services [36]. Thus, appropriate use is necessary to prevent the emergence of drug resistant bacteria and minimize unnecessary wastage of scarce resource [37].

Overuse of injections is also one form of irrational use of drugs. In developing countries, up to 56% of primary-care patients receive injections where over 90% may be medically unnecessary [26]. In this study, the percentage of encounters with injection (13.9%) was within the WHO standard range of 13.4–24.1% [5]. It was comparable with the finding of a study conducted in North East Ethiopia (13.8%) [30]. The finding was lower than reports from Nekemete health center (21.94%) [16], South Ethiopia (38.1%) [12], North Ethiopia (42.2%) [17], East Ethiopia (34.6%) [28], North West Ethiopia (48.36%) [15], Sierra Leone (26%) [32], Kenya (24.9%) [11] and Pakistan (27.1%) [33]. However, the finding was higher than encounter with injection in West Shoa zone (12.6%) [29], public hospital of Eastern Ethiopia (10.9%) [31], Zambia (9.7%) [10], Eritrea (7.8%) [35] and Egypt (9.9%) [34]. The use of unsterilized needle and syringe may result in the transmission of serious infectious diseases [26]. Therefore, an urgent need arises to reduce injection used to prevent healthcare-associated infections, and safe, cost-effective, and simple oral alternatives should be promoted.

Drug prescribing using generic name rationalize drug therapy and minimize the cost of treatment. In this study, we observed that 98% of drugs were prescribed by generic name. This finding compares with previous findings from WHO [5], Ethiopia [13, 30, 31], and Egypt [34]. The percentage of generic prescription was higher compared to other studies from Ethiopia [12, 15, 17, 29], Eritrea [35], Sierra Leone [32], Kenya [11], Zambia (9.7%)[10], and Pakistan [33]. Generic prescription is an indicator of prescribing quality which can reduce the cost of prescribed medications and can determine the level of compliance [36, 38].

The percentage of drugs from the essential drug list was 100% which was agreed with the WHO standard [5, 36]. This finding was higher than studies from Ethiopia [12, 15, 17, 28, 29], Eritrea [35], Sierra Leone [32], Pakistan [33], Zambia (9.7%) [10], and Kenya [11]. The concept of essential medicine is relevant to health programs for better use of health resources, good therapeutics outcomes, and reduced side-effects of medicines. Rational drug use is achieved when there is a rational prescribing using medicine from an EDL. Identifying a limited number of essential medicines leads to better supply, more rational use, and lower costs. Drugs in the EDL are intended to satisfy the health needs of the majority of the population in diagnostic, prophylactic, therapeutic, and rehabilitative services [38].

In this study, the average consultation time was 4.7 min which was lower than 10 min of WHO standard [5, 26]. Higher average consultation time was reported in the West Shoa zone (5.12 min) [29], North West Ethiopia (10.46 min) [13], Kenya (4.1 min) [11], and Pakistan (2.2 min) [33]. It was better than studies conducted in North East Ethiopia (1.57 min) [30], Eastern Ethiopia (4.61 min) [28], Egypt (7.1 min) [34]. The average dispensing time (105 s) was lower than the WHO standard value of 180 s or more, but better than studies from Ethiopia (47 s) [15], (43.69 s) [15], and (61.12 s) [15], and Egypt (47.4 s) [34]. However, it was still lower than studies in Ethiopia (108 s) [30] and Kenya (131.5 s) [11]. Pharmacists and physicians are highly visible and readily available health professionals to solve patients' concerns, have the responsibility to safeguard the patients' health and ensure the success of the drug therapy by providing appropriate counseling to maximize desired therapeutic outcomes [38, 39].

The percentage of drugs actually dispensed was 92%, and none of the drugs were adequately labeled. The value was lower than the WHO standard (100%) [5, 26]. Despite the percentage of drugs actually dispensed was better than findings from Ethiopia (82.6%) [30], (73.2%) [29], Kenya (76.3%) [11] and Pakistan (90.9%) [33], lower than Ethiopia (96.17%) [15], (100%) [31], and Egypt (95.9%) [34]. The percentage of drugs that were adequately labeled was in line with studies from Ethiopia [29] and Egypt [34]. But lower than other studies conducted in Ethiopia (20%) [31], (22.7%) [30], Kenya (22.6%) [11] and Pakistan (100%) [33]. The percentage availability of the WHO model list of key essential medicines was 64.1%. Higher percentage availability of the WHO model list of key essential medicines was reported from Ethiopia (93.75%) [15], Pakistan (100%) [33], and Egypt (78.3%) [34]. The shortage of key essential medicines may be related to the ineffective use of resources and poor inventory management systems [25, 39].

The WHO recommended that all (100%) of patients must have adequate knowledge of their medication [5, 26]. In this study, the percentage of patients who had adequate knowledge was 54.8%. This finding compares with previous findings from Kenya (54.7%) [11]. The percentage of patient knowledge was lower according to other studies from Ethiopia (74.67%) [30], (69.6%) [29], (75.5%) [31], Egypt (95%) [34] and Pakistan (62.9%) [33]. However, other studies also report much lower knowledge than what we observed from Ethiopia (5.83%) [15]. The difference might be due to educational background, cultural factors, physical factors such as noises, and short dispensing time. Limited patient knowledge of their medication will worse health outcomes and may result in increased mortality [5].

The present study revealed that the overall IRDP was 4.43. It was lower than the ideal of 5. However, the IRDP was higher than the finding of a study from Sierra Leone (2.6) [32]. In the Bahawalpur district of Pakistan [33], the Eastern province of Saudi Arabia [9], and in Egypt [34], the IRDPs ranged from 3.78 to 4.27, 3.77 to 5, and 3.92 to 4.88 respectively. The result of IRDP indicated that the prescribing practice should be improved more.

The findings of the drug use indicator will serve as a first-line measure which is intended to stimulate further questioning and guide subsequent action. Moreover, it will help to improve the performance of health professionals and health facilities in maximizing therapy. Thus, health managers can intervene in process improvement and service delivery performance. Inclusion of few number of health facilities might be the limitation of the present study; because increasing the number of facilities in the sample is recommended to obtain a more accurate and reliable estimates of overall prescribing practices [26].

## Conclusion

This study demonstrated irrational drug use practices in all healthcare facilities. Polypharmacy, antibiotics over-prescribing, short consultation and dispensing times, poor labeling of medicines, inadequate level of patients' knowledge about prescribed medicines, and unavailability of key drugs in stock were found to be the major problems. Continuous refreshment trainings on rational use of drugs and WHO recommendations should be given for prescribers and pharmacists. Further, we recommend studies involving large number of facilities to estimate overall prescribing practices.

## Data Availability

The datasets used and/or analyzed during the current study are available from the corresponding author on reasonable request.

## Abbreviations

EDL: Essential drug list
INRUD: International network for rational use of drugs
IRDP: Index of Rational Drug Prescribing
IRPCDU: Index of Rational Patient-Care Drug Use
IRFSDU: Index of Rational Facility- Specific Drug Use
IRDU: Index of Rational Drug Use
STG: Standard treatment guideline
WHO: World Health Organization
